# Auxin as an architect of the pectin matrix

**DOI:** 10.1093/jxb/erad174

**Published:** 2023-05-11

**Authors:** François Jobert, Sandeep Yadav, Stéphanie Robert

**Affiliations:** Umeå Plant Science Centre, Department of Forest Genetics and Plant Physiology, Swedish University of Agricultural Sciences (SLU), 90183, Umeå, Sweden; CRRBM, Université de Picardie Jules Verne, 80000, Amiens, France; Umeå Plant Science Centre, Department of Forest Genetics and Plant Physiology, Swedish University of Agricultural Sciences (SLU), 90183, Umeå, Sweden; Umeå Plant Science Centre, Department of Forest Genetics and Plant Physiology, Swedish University of Agricultural Sciences (SLU), 90183, Umeå, Sweden; MPI of Molecular Plant Physiology, Germany

**Keywords:** Auxin, calcium (Ca^2+^), cell wall, microdomains, pectin, pectin methylesterase, pH

## Abstract

Auxin is a versatile plant growth regulator that triggers multiple signalling pathways at different spatial and temporal resolutions. A plant cell is surrounded by the cell wall, a complex and dynamic network of polysaccharides. The cell wall needs to be rigid to provide mechanical support and protection and highly flexible to allow cell growth and shape acquisition. The modification of the pectin components, among other processes, is a mechanism by which auxin activity alters the mechanical properties of the cell wall. Auxin signalling precisely controls the transcriptional output of several genes encoding pectin remodelling enzymes, their local activity, pectin deposition, and modulation in different developmental contexts. This review examines the mechanism of auxin activity in regulating pectin chemistry at organ, cellular, and subcellular levels across diverse plant species. Moreover, we ask questions that remain to be addressed to fully understand the interplay between auxin and pectin in plant growth and development.

## Teaser

The phytohormone auxin regulates most aspects of plant development. Auxin activity leads to intense modifications of the primary cell wall’s pectin matrix, which we will focus on in this review.

## Introduction

Auxin plays a crucial role in plant growth and morphogenesis, controlling the balance between cell division and elongation, and integrating environmental cues (Leyser, 2018). The primary cell wall, which restricts plant cell expansion, is composed mainly of polysaccharides, which are long chains of sugars, such as cellulose, hemicellulose, and pectin, as well as proteins. The interactions between different polysaccharides in the cell wall are complex and dynamic, and changes in their composition or arrangement can alter the mechanical properties of the cell wall (Sarkar *et al.*, 2009). Cellulose is a linear polymer of glucose molecules that form long chains held together by hydrogen bonds to form a rigid, crystalline structure (Wohlert *et al.*, 2022). Pectin, a negatively charged polysaccharide, modulates cell wall mechanical properties by forming hydrogels and through interactions with other cell wall components (Voragen *et al.*, 2009; Cosgrove, 2022). A plethora of enzymes play key roles in reorganizing the pectin backbone, allowing cell expansion through turgor-driven pressure from the vacuole (Mohnen, 2008). Auxin regulates the expression of numerous genes responsible for cell wall reorganization, and its effect on cell rigidity and growth is dependent on the developmental stage, tissue type, and organ (Majda and Robert, 2018).

The ‘acid growth theory’ posits that auxin induces cell elongation by acidifying the apoplast and thereby activating cell wall loosening enzymes in shoots (Rayle and Cleland, 1970; Hager *et al.*, 1971), but the effect of auxin on apoplastic pH in roots is less clear and auxin may actually inhibit root cell expansion. Recent discoveries have shed light on the molecular mechanisms by which auxin can modulate the pH of cell wall compartments in root, but it is still uncertain how this affects the structure and mechanics of cell wall pectin (Fendrych *et al.*, 2016, 2018; Barbez *et al.*, 2017; Li *et al.*, 2021; Lin *et al.*, 2021). This review is an update on the recent advances concerning the connection between auxin and pectin modulation in the cell wall during plant growth and development. We first provide an overview of cell wall pectin and discuss recent discoveries related to auxin, before delving into the role of auxin in regulating pH and calcium (Ca^2+^) levels, and how this influences the formation of pectin ‘microdomains’ with varying compositions (Dauphin *et al.*, 2022). Finally, we discuss auxin-mediated transcriptional regulation of genes responsible for pectin modification and how this process may involve feedback mechanisms that help to maintain a delicate balance in plant growth.

## Cell wall pectin at a glance

Pectins are complex heteropolysaccharides making up roughly 30% of the primary cell wall in plants (Cosgrove, 2022). Pectin structure is diverse and based on four kinds of domains. Linear homogalacturonans (HG) are the most abundant and consist of α-(1-4)-linked d-galacturonic acid (GalA) linear chains where carboxyl groups can be methylesterified or acetylesterified (Caffall and Mohnen, 2009). The branched rhamnogalacturonan-I (RG-I) backbone is composed of repeating disaccharide units of GalA and α-(1-2)-l-rhamnose with side chains containing galactans, arabinans, and/or arabinogalactans. RG-II is the smallest but most complex domain. It is composed of a GalA backbone substituted by four chains containing more than 10 different sugars. Finally, xylogalacturonans are less common and are based on a β-1,4-linked glucan backbone that is decorated with a GalA chain and d-xylose residues.

Several carbohydrate-active enzyme families are involved in the biosynthesis, modification, and degradation of pectin. Polymerization and branching of the different pectin components are catalysed by glycosyltransferases while their degradation is mediated by glycosylhydrolases (Minic and Jouanin, 2006; Atmodjo *et al.*, 2013). The HG backbone is secreted into the wall in a highly methylesterified form (Zhang and Staehelin, 1992). Modification of pectins can occur *in muro* through enzymes containing a broad spectrum of pH-dependent activities, making the pectin network dynamic. The stability of pectins is affected by the presence or absence of Ca^2+^ and borate, which can form ‘egg-box’ structures between demethylesterified HG and borate diester crosslinks in the case of RG-II polysaccharides (Cosgrove, 2022). Variations in apoplastic pH and ion movements across membranes inevitably impact the pectin network by altering the strength of intermolecular interactions and modifying enzyme activity (Haas *et al.*, 2021). Pectin distribution, which can be heterogeneous along and across the wall of individual cells, plays a crucial role in regulating cell wall mechanical properties, and such heterogeneity has been linked to epidermal cell shape acquisition (Majda *et al.*, 2017), xylem fibre elongation (Majda *et al.*, 2021), pollen tube and root hair cell elongation (Dardelle *et al.*, 2010; Chebli *et al.*, 2012), and stomatal aperture (Amsbury *et al.*, 2016). Thus, the precise distribution and composition of pectin within the cell wall is crucial for proper plant morphogenesis.

## A quick response: revisiting the acid growth theory

**Fig. 1.**
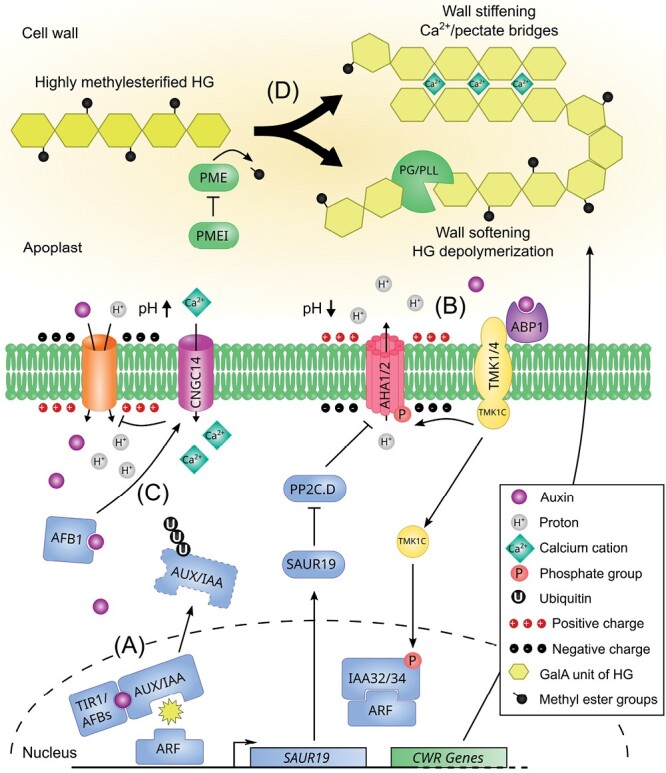
An updated view of the auxin-triggered signalling cascades. (A) Nuclear auxin signalling pathway. Auxin is perceived intracellularly by the TIR1/AFB–AUX/IAA co-receptor complex, resulting in the polyubiquitinylation and degradation of AUX/IAAs, which releases the inhibition of the ARF transcription factors in the nucleus. This triggers the expression of auxin responsive genes, among which *SAUR19* whose protein inhibits the phosphatase PP2C.D, preventing the deactivation of the plasma membrane H^+^-ATPase AHA1/2 through dephosphorylation of the penultimate residue. This cascade results in gradual apoplast acidification and hyperpolarization of the plasma membrane. In the meantime, auxin regulates the expression of cell wall remodelling enzymes modulating pectin properties (see (D)). (B) TMK-based auxin extracellular signalling pathway. Auxin is perceived in the apoplast by the secreted receptor ABP1 and its plasma membrane partners from the TMK family. This activates the TMKs, which in turn phosphorylate AHA1/2, allowing a net efflux of H^+^ and the rapid hyperpolarization of the plasma membrane. In the context of apical hook growth cessation, the TMK1 C-terminal domain is processed by an unknown molecular actor and translocates to the nucleus where it phosphorylates and stabilizes the non-canonical IAA32/34 repressors. (C) TIR1/AFB auxin extranuclear signalling pathway. Auxin enters the cell via AUX1-based IAA–H^+^ symport, triggering rapid plasma membrane alkalinization and depolarization. IAA perception by the cytoplasmic AFB1 complex elicits Ca^2+^ influx dependent on CNGC14. The high [Ca^2+^]_cyt_ inhibits AUX1-mediated IAA influx in a negative feedback loop through an unknown mechanism. (D) The main pectin component, homogalacturonan, is secreted into the cell wall in a highly methylesterified form. Demethylesterification is catalysed by PME enzymes, which are actively regulated by PME proteinaceous inhibitors. Depending on the PME processivity, blockwise demethylesterified HG galacturonic acid chains can form Ca^2+^/pectate bridges and organize into an ‘egg-box’-like structure thought to stiffen the cell wall. In contrast, non-blockwise or random demethylesterification results in HG depolymerization through pectinolytic enzyme activity (PG/PLL) and softening of the cell wall.

The acid growth theory was first proposed by Rayle, Cleland, Hager, and colleagues (Rayle and Cleland, 1970; Hager *et al.*, 1971) and was based on the observation of rapid elongation of *Avena* hypocotyls and *Helianthus* coleoptiles when grown in acidic buffers. The theory proposes that the presence of auxin in a plant cell stimulates the activity of membrane-bound H^+^-ATPase proton pumps, leading to an influx of protons (H^+^) into the cell wall. This leads to a decrease in apoplastic pH, which generates an acidic environment that activates enzymes to loosen the wall, thus allowing cell elongation. Two mechanisms by which auxin can activate plasma membrane H^+^-ATPase have now been uncovered (Fig. 1A, B). The first requires the canonical nuclear auxin signalling pathway (Fig. 1A) (Salehin *et al.*, 2015). Upon binding of auxin to TRANSPORT INHIBITOR RESPONSE1/AUXIN-SIGNALING F-BOX (TIR1/AFB) receptor proteins, TIR1/AFB undergoes a conformational change that enables it to interact with AUXIN/INDOLE-3-ACETIC ACID (AUX/IAA) transcriptional repressor proteins. Recognition of AUX/IAA proteins by the SCF^TIR1/AFB^ complex leads to the polyubiquitination of AUX/IAA and its subsequent degradation via the 26S proteasome, alleviating suppression of class A AUXIN RESPONSE FACTOR (ARF) transcription factors, which can now modulate the expression of downstream genes (Chapman and Estelle, 2009; Cancé *et al.*, 2022). ARFs bind to specific DNA sequences called auxin response elements (AuxREs) located in the promoter regions of target genes, thereby regulating their expression (Leyser, 2018). Auxin induces the transcription of *SMALL AUXIN UP RNA19* (*SAUR19*), leading to the activation of plasma membrane localized H^+^-ATPase by promoting phosphorylation of its C-terminal autoinhibitory domain. Furthermore, PROTEIN PHOSPHATASE 2C.D (PP2C.D) interacts with SAUR19 and negatively regulates H^+^-ATPase activity (Takahashi *et al.*, 2012; Spartz *et al.*, 2014).

A second auxin response, occurring within a minute, potentially involves extracellular auxin perception by secreted AUXIN BINDING PROTEIN 1 (ABP1)/ABP-LIKE1 and 2 (ABL1/2) and its interaction with the co-receptor TRANSMEMBRANE KINASE1/4 (TMK1/4) (Fig. 1B) (Friml *et al.*, 2022; Yu *et al.*, 2022, Preprint). TMK then phosphorylates ARABIDOPSIS H^+^-ATPase 1 (AHA1) and initiates its activation, leading to apoplast acidification and rapid cell expansion in shoot tissue (Lin *et al.*, 2021). TMKs are part of a signalling hub, possibly involving other kinases, where the auxin-induced phospho-response of thousands of proteins occurs after 30 s of IAA treatment (Roosjen *et al.*, 2022, Preprint). Among the most notable targets are the polar auxin efflux transporters PIN-FORMED 1 (PIN1) and PIN2, whose phosphorylation status regulates their cellular localization (Zhang *et al.*, 2010; Rodriguez *et al.*, 2022, Preprint; Wang *et al.*, 2022, Preprint). In addition, auxin promotes the cleavage of the TMK1 C-terminus in a TIR1/AFB-independent manner. The TMK1 C-terminal domain translocates to the nucleus where it interacts with and stabilizes IAA32/34, affecting the auxin transcriptional machinery (Cao *et al.*, 2019). To conclude, we can say that auxin triggers significant acidification of the cell wall in the hypocotyl and possibly in other parts of the shoot. However, in the root, the initial response to auxin is actually an increase in alkalinity, implicating the existence of diverse and intricate mechanisms in various parts of the plant. Despite activating AHA1 through TMK-mediated phosphorylation, auxin arrests primary root growth within minutes (Monshausen *et al.*, 2011; Li *et al.*, 2021). This physiological effect is preceded by apoplast alkalinization and relies on the TIR1/AFB module, with the AFB1 paralogue playing a crucial role, but not on nuclear auxin signalling components (Barbez *et al.*, 2017; Fendrych *et al.*, 2018; Serre *et al.*, 2021; Dubey *et al.*, 2023, Preprint) (Fig. 1C). The TIR1/AFB downstream molecular mechanisms fully explaining apoplast alkalinization are still unclear. Auxin influx transporter AUXIN RESISTANT 1 (AUX1) can partially mediate IAA–H^+^ symport and proton uptake, as the *aux1* mutant remains slightly sensitive to auxin-mediated alkalinization (Monshausen *et al.*, 2011; Dindas *et al.*, 2018). In addition, AUX1 plays a key role in the establishment of an alkaline surface domain in the root transition zone (Serre *et al.*, 2022, Preprint). The receptor-like kinase FERONIA (FER) triggers apoplastic alkalinization upon binding of its ligand RAPID ALKALINIZATION FACTOR 1 (RALF1) (Haruta *et al.*, 2014). During the gravitropic root response, FER modulates cell expansion while auxin controls the onset of cellular elongation through the regulation of apoplastic acidification (Barbez *et al.*, 2017). Activation of plasma membrane H^+^-ATPases in roots occurs through phosphorylation of cell surface TMK1-based auxin signalling, resulting in apoplast acidification, which acts in opposition to the dominant TIR1/AFB-mediated alkalization (Li *et al.*, 2021). Further, it is proposed that TIR1/AFB-mediated apoplastic alkalization does not require FER, as *fer-4* mutants display normal auxin-induced rapid growth inhibition (Li *et al.*, 2021). So, it remains to be clarified how FER contributes to apoplast acidification.

Interestingly, AFB1-mediated inhibition of root growth is also preceded by membrane depolarization and cellular Ca^2+^ intake (Serre *et al.*, 2021, 2022, Preprint; Li *et al.*, 2021). The activation of the CYCLIC NUCLEOTIDE GATED CHANNEL 14 (CNGC14) Ca^2+^ channel requires operational TIR1/AFB auxin-based perception (Monshausen *et al.*, 2011; Shih *et al.*, 2015; Dindas *et al.*, 2018). Strikingly, high cytosolic Ca^2+^ concentration or blocking CNGC14 inhibits AUX1 activity, suggesting the existence of a Ca^2+^-dependent negative feedback loop in the AUX1–TIR1/AFB–CNGC14 module (Dindas *et al.*, 2018). Regardless of the speed of the response, it appears clear that both nuclear and extranuclear auxin signalling pathways play roles in cell wall modulation (Hocq *et al.*, 2017a; Majda and Robert, 2018).

## Auxin-induced apoplast pH variations regulate pectin chemistry or structure

The incubation of plant organs in acidic buffers quickly triggers cell elongation (Barbez *et al.*, 2017; Li *et al.*, 2021). Auxin induces striking cell wall acidification in the shoot, but in the root the first effect of auxin observed is actually alkalinization, suggesting the existence of complex and varied mechanisms in different parts of the plant. While plant cells have a wide range of pH values ranging from 3.5 to 8.3 (Yu *et al.*, 2000; Moreau *et al.*, 2021; Tsai and Schmidt, 2021), the apoplast is generally acidic, with pH values ranging from 4 to 6.3 and decreasing towards the plasma membrane (Martinière *et al.*, 2018). Among several other factors, the apoplastic pH plays a major role in determining the optimal activity of cell wall and pectin remodelling enzymes. The degree of methylesterification is one of the most studied pectin modifications and can be controlled by both PECTIN METHYLESTERASES (PMEs) and their inhibitors (PMEIs) (Fig. 1D; Sénéchal *et al.*, 2014). While plant PMEs usually show increased activity in alkaline buffers (Dixit *et al.*, 2013; Del Corpo *et al.*, 2020; Hocq *et al.*, 2021, Preprint), their inhibition by PMEIs is more effective under acidic conditions (Sénéchal *et al.*, 2015; Bonavita *et al.*, 2016; Hocq *et al.*, 2017b; Xu *et al.*, 2022a). This can be explained by the formation of a pH-dependent reversible protein complex comprising both a PME and PMEI. For example, the Arabidopsis PME3 and PMEI7 complex is stable at a low pH, depending on the protonation state of amino acid residues at the binding interface (Sénéchal *et al.*, 2017). Changes in pH can regulate blockwise PME processivity, with full processivity occurring in slightly alkaline conditions and partial processivity in acidic conditions (Hocq *et al.*, 2021, Preprint). The pH-dependent fine-tuning of PME processivity can affect the mechanical properties of the cell wall by forming stiffening pectate–Ca^2+^ structures or triggers substrate availability for pectin degrading enzyme (Fig. 1D). In addition to the pH microenvironment, the degree of pectin methylesterification can also be fine-tuned by the stoichiometry of PMEs and PMEIs, and specific residues in their docking sites (Sénéchal *et al.*, 2017; Hocq *et al.*, 2017b). These findings highlight the complexity of the mechanisms involved in pectin modification and underscore the importance of understanding the role of pH in regulating these processes.

PME enzymes catalyse the de-esterification of methylesterified HG, resulting in the release of demethylesterified HG, methanol, and protons. The released protons potentially contribute to apoplast acidification in the direct microenvironment (Hocq *et al.*, 2017a). According to their pH optimum, this could set up a negative feedback loop wherein a PME is locally inactivated by a PMEI in acidic conditions. Interestingly, pectin degrading enzymes, such as polygalacturonases (PGs), have been reported to act preferentially on demethylesterified substrates at a slightly acidic pH (<5.5) (Hocq *et al.*, 2020; Ohashi *et al.*, 2022), typically in the range of the apoplastic pH observed during auxin-induced acidification.

Despite recent advances in measuring wall mechanical properties using atomic force microscopy and Brillouin microscopy (Bacete *et al.*, 2022), the consequences of PME processing for pectin stiffness remain unclear. For example, the induction of *PME5* expression in Arabidopsis resulted in HG demethylesterification, which caused an increase or decrease in wall stiffness in dark-grown hypocotyl, depending on the study (Peaucelle *et al.*, 2015; Bou-Daher *et al.*, 2018). In the shoot apical meristem, the expression of *PME5* led to a softer cell wall (Peaucelle *et al.*, 2011). Gallemí *et al.* (2022, Preprint) proposed that the discrepancies observed between experiments might be attributed to differences in the inducible line used or the duration of transgene induction, which might influence the accumulation of the induced protein. *PME1* and *PME5* induction for a short time generated a stiffer cell wall, while a sustained induction resulted in a softer cell wall. Periodic and/or moderate PME activity likely produces low methylesterified HG (blockwise demethylesterification), which is able to form Ca^2+^ cross-links, strengthening the pectin structural network. On the other hand, continuous and/or high PME activity generates long stretches of demethylesterified HG that could be degraded by hydrolysing enzymes (Gallemí *et al.*, 2022, Preprint). This prolonged action of PME could release a substantial amount of H^+^ and that could further acidify the apoplast, activating other cell wall actors such as expansins to induce cell wall loosening (Cosgrove, 2022).

## When Ca^2+^ enters the pectin matrix

The ability of demethylesterified HG to interact with divalent cations to form ‘egg-box’ structures affects the cell wall mechanical properties. Additionally, Ca^2+^ is also an important second messenger in many cellular signalling cascades and Ca^2+^ waves are an important feature of the rapid auxin response (Vanneste and Friml, 2013). In roots, cytosolic Ca^2+^ concentration ([Ca^2+^]_cyt_) increases within seconds after auxin treatment (Monshausen *et al.*, 2011), an effect mediated by the AUX1–TIR1/AFB–CNGC14 module (Fig. 1, Shih *et al.*, 2015; Dindas *et al.*, 2018; Serre *et al.*, 2022, Preprint). Apoplastic Ca^2+^ concentration ([Ca^2+^]_apo_) usually ranges between 10 µM and 10 mM while [Ca^2+^]_cyt_ is much lower, at around 100 nM (Hepler and Winship, 2010). For auxin-induced Ca^2+^ influx to occur in the cytosol, the presence of available Ca^2+^ for transport is a prerequisite, and this process also requires the activity of CNGC14. It has been shown that auxin-mediated rapid root growth inhibition in maize requires Ca^2+^ ions and their availability limits the auxin effect (Hasenstein and Evans, 1986). Ca^2+^ is substantially stored in vacuoles where the CATION EXCHANGER 1/3 (CAX1/3) Ca^2+^/H^+^ exchangers finely regulate pH gradients and Ca^2+^ transients during stomatal closure (Conn *et al.*, 2011; Cho *et al.*, 2012). The *cax1/3* double mutant displays constitutive plasma membrane hypopolarization and auxin insensitivity during abscisic acid-induced stomatal closure, which can be rescued by lowering the apoplastic pH (Cho *et al.*, 2012). Curiously, AUX1 pharmacological inhibition phenocopied *cax1/3*, and *cax1* root phenotypes resemble that of the *aux1* mutant (Cheng *et al.*, 2003; Cho *et al.*, 2012). These results suggest a requirement for vacuolar Ca^2+^/H^+^ exchangers in the proper regulation of ion and pH homeostasis between the different cell compartments.

The cell wall also acts as an important source of Ca^2+^ for the plant cell. It can be nicely depicted in the growing pollen tube tip region, where demethylesterification through PME leads to the release of Ca^2+^ in the apoplastic region of the cell wall. PME activity can facilitate the formation of Ca^2+^ pectate bridges between blockwise demethylesterified HG galacturonic acid chains, resulting in the arrangement of these chains into an ‘egg-box’-like structure to stiffen the cell wall (Hepler *et al.*, 2012). Interestingly, guard cell walls are depleted in Ca^2+^-crosslinked HG, and PME activity is needed for stomatal closure (Amsbury *et al.*, 2016). The addition of EGTA is known to stimulate expansin-mediated cell growth by depleting the [Ca^2+^]_apo_ (Zhao *et al.*, 2008). Auxin-mediated cell wall softening and hypocotyl elongation are limited by the addition of Ca^2+^ to the growth medium (Gallemí *et al.*, 2022, Preprint). Interestingly, the addition of Ca^2+^ enhances the stiffening of onion epidermal walls by treatment with PME (Wang *et al.*, 2020). This synergistic effect with PME is also seen in Arabidopsis overexpressing *PME1* for a short period of time, where the addition of the Ca^2+^ chelator EGTA suppresses the stiff cell wall phenotype (Gallemí *et al.*, 2022, Preprint). Overall, these results question the notion that the structure of the pectin matrix is the sole determinant of any growth effects mediated by auxin.

The visualization of the Ca^2+^–pectate structure has been inferred widely by using the antibody 2F4, but this method requires high [Ca^2+^]. Mravec *et al.* (2017) demonstrated real time imaging of calcium mediated crosslinking of HG *in muro* using the fluorescently tagged long oligogalacturonides probe OG7-13. Usage of OG7-13 might better help us to understand the importance of auxin in the formation of pectin ‘egg-box’ structures *in planta*. Despite the availability of tools such as the genetically encoded Förster resonance energy transfer-based Ca^2+^ sensor Yellow Cameleon 3.60 (Nagai *et al.*, 2004; Monshausen *et al.*, 2011) or the single fluorophore R-GECO1 (Zhao *et al.*, 2011; Keinath *et al.*, 2015), it remains challenging to observe and measure changes in [Ca^2+^] at the subcellular resolution in the context of rapid auxin growth responses, owing to its highly dynamic fluctuations. The sensor family from the CamelliA toolbox and its plasma membrane-anchored version might help to uncover Ca^2+^ dynamics at the apoplast–cytosol interface (Guo *et al.*, 2022). While it is known that high [Ca^2+^]_cyt_ can induce the expression of pectin remodelling enzyme genes (Conn *et al.*, 2011; Wang *et al.*, 2015), the mechanism underlying the connection between this phenomenon and auxin-mediated Ca^2+^ entry remains unclear. Downstream signalling components might be identified in the future that with the help of pharmacological inhibitors of auxin-induced Ca^2+^ signalling may improve our understanding (De Vriese *et al.*, 2019).

## The spatial control of pectin remodelling

The feedback loop between chemical instructions and mechanical changes for pattern formation in development has recently gained significant interest. Auxin reduces tissue rigidity in the shoot apex of Arabidopsis before organ outgrowth by demethylesterifying pectin, and the development of functional organs requires auxin signalling (Braybrook and Peaucelle, 2013). Also, cell wall rigidity affects the localization of PIN1 in the shoot apex, suggesting a feedback loop between auxin and cell wall mechanics in phyllotactic patterning (Braybrook and Peaucelle, 2013). The pleiotropic effects of auxin are highly dependent on organ sensitivity and the developmental stage under investigation. As we previously mentioned, auxin tends to rapidly promote cell elongation in the shoot whereas it quickly represses cell expansion in the root (Fendrych *et al.*, 2016, 2018; Barbez *et al.*, 2017; Li *et al.*, 2021; Lin *et al.*, 2021).

Many insights have been gained from studying organs that exhibit differential growth, where cells present on one side elongate more than those on the other side. One such example of this is apical hook formation, which involves the bending of the hypocotyl upon germination to protect the shoot apical meristem from mechanical damage. Auxin signalling plays a key role in the regulation of this process (Béziat and Kleine-Vehn, 2018). Asymmetric distribution of auxin, via polar localization of auxin transporters, controls the differential degree of methylesterification of pectins between the inner and outer side of the apical hook (Jonsson *et al.*, 2021). Indeed, cells located on the inner side of the hook display a strong auxin response and a higher degree of methylesterification than cells located on the outer side, which is correlated with a reduction in cell deformation and elongation (Jonsson *et al.*, 2021; Du *et al.*, 2022). It is noteworthy that plants overexpressing *PMEI5*, with a uniformly high degree of pectin methylesterification, fail to establish the proper auxin response gradient, which results in the failure of apical hook formation. These observations suggest the existence of a positive feedback loop between pectin demethylesterification and auxin signalling (Jonsson *et al.*, 2021). Gravistimulation induces differential cell growth in the root elongation zone, and auxin plays a dual role in promoting and inhibiting cell elongation on the upper and lower sides of the bending root, respectively. Despite our deep understanding of the auxin role in this model (reviewed in Konstantinova *et al.*, 2021), the dynamics of the cell wall and, in particular, pectin, remain poorly understood. Further research is needed to understand how the complex interactions between cell wall components and other cellular components lay the foundation for differential cell growth.

Auxin and pectin remodelling are also tightly intertwined in other physiological processes such as cell adhesion, where temporal and spatial regulations are critical for the maintenance of cell-to-cell contacts, which are essential for the integrity and function of tissues and organs. During lateral root emergence, the cells located in front of the growing lateral root primordium undergo extensive cell wall modifications. Auxin leaks from the primordium tip and is funnelled into the overlying cells through the activation of the auxin influx carrier LIKE AUX1 3 (LAX3) in a positive feedback loop (Swarup *et al.*, 2008; Péret *et al.*, 2013). Auxin induces the local expression of *INFLORESCENCE DEFICIENT IN ABSCISSION* (*IDA*), which encodes a peptide that then binds to its receptor, HAESA/HAESA-LIKE2 (HAE/HSL2), to trigger a signalling cascade that activates a transcriptional amplification mechanism for pectin remodelling genes. This in turn affects cell wall properties and ultimately influences cell growth and tissue morphogenesis (Kumpf *et al.*, 2013). Intriguingly, low methylesterified pectins were found to be reduced at the site of lateral root emergence, at the pericycle–endodermis and endodermis–cortex junctions, as revealed via immunolabelling using the LM19 antibody (Wachsman *et al.*, 2020). This result seems counterintuitive with the prevailing model, which suggests that demethylesterification of pectins serves to prime the substrate for pectin-degrading enzyme-encoding genes such as *POLYGALACTURONASE INVOLVED IN LATERAL ROOT* (*PGLR*), which are expressed in those cells (Kumpf *et al.*, 2013; Hocq *et al.*, 2020). Instead, the authors propose that the loss of demethylesterified pectins may lead to a reduction in cell adhesion (Wachsman *et al.*, 2020). Auxin’s positive effect on secondary root emergence seems to be largely conserved in plants. A recent study using white lupin (*Lupinus albus*) as an alternative model showed that auxin triggers pectin demethylesterification in the rootlet emergence zone (Jobert *et al.*, 2022b). Similarly to Arabidopsis primordium overlying cells, auxin induces the expression of *LaPG3* in the rootlet emergence zone of white lupin, which is a close homologue of *PGLR* and predicted to act on demethylesterified pectins (Jobert *et al.*, 2022b).

Cell separation occurs in floral organs and fruits, where the balance between auxin and ethylene coordinates abscission (Basu *et al.*, 2013; Gao *et al.*, 2019; Dong *et al.*, 2021). Auxin transport plays a pivotal role in determining the abscission zone in Arabidopsis mature siliques, yellow lupin flowers, and tomato fruits (Sorefan *et al.*, 2009; Kućko *et al.*, 2020; Dong *et al.*, 2021). The IDA–HAE/HSL2 module plays a conserved role in controlling organ shedding in many species by upregulating *PG* genes (Aalen *et al.*, 2013). Furthermore, a recent study using Arabidopsis floral cell sorting combined with single cell RNAseq identified the expression of pectin remodelling genes regulated by HAE/HSL2 in the abscission zone (Taylor *et al.*, 2022, Preprint). While it is unclear whether auxin is also involved in IDA–HAE/HSL2 signalling cascade activation during lateral root emergence, the expression of *POLYGALACTURONASE ABSCISSION ZONE A. THALIANA* (*PGAZAT*) and *PGLR* in cells overlying lateral root primordia and in abscission zones imply the presence of a shared regulatory module (Kumpf *et al.*, 2013). Moreover, this suggests that different tissues can have similar HG composition that could represent a possible hallmark for the breakdown of the middle lamella and, ultimately, cell separation. In the oil palm, immunolocalization and Fourier-transform infrared spectroscopy experiments revealed highly demethylesterified pectin content in the separated abscission zone cell layer surface (Roongsattham *et al.*, 2016). The acquisition of cell polarity for local pectin modification and degradation implies the formation of wall regional territories. This exciting research field has been extensively reviewed recently (Raggi *et al.*, 2020; Dauphin *et al.*, 2022). Thus, the interplay between auxin and pectin dynamics is a fundamental aspect of plant development and physiology, with broad implications for plant adaptation and survival.

## How auxin defines cell wall microdomains

Cell wall microdomains refer to specific regions of the plant cell wall that possess distinct compositions and organizations of wall polymers, enabling the plant to undergo localized processes of wall softening or stiffening, which are essential for its growth and development (Dauphin *et al.*, 2022). The clustering of PIN proteins and its impact on polar auxin transport is influenced not only by the connections between the plasma membrane and the cell wall, but also by the composition of the cell wall itself (Feraru *et al.*, 2011; Kleine-Vehn *et al.*, 2011; Ke *et al.*, 2021; Marhava, 2022). Specifically, the composition of pectin and cellulose within the cell wall affects the size, density, and diffusion rate of PIN clusters (McKenna *et al.*, 2019; Ke *et al.*, 2021). This suggests that dynamic changes in the cell wall could provide feedback cues to alter auxin gradient formation by changing PIN polarity in the plasma membrane.

Intracellular wall heterogeneity in regards to pectin chemistry has been observed in several developmental contexts including hypocotyl anisotropic growth (Bou Daher *et al.*, 2018), pavement cell lobe initiation and growth (Majda *et al.*, 2017; Altartouri *et al.*, 2019), pollen tube and root hair bulging/elongation (Chebli *et al.*, 2012; Mravec *et al.*, 2017; Schoenaers *et al.*, 2018), stomatal closure (Amsbury *et al.*, 2016), seed mucilage secretory cell differentiation (Francoz *et al.*, 2019), and phloem sieve element maturation (Kalmbach *et al.*, 2023). Distinct pectin polymer signatures and pectin modifying enzymes are also found in the vicinity of plasmodesmata. Plasmodesmata cell wall microdomains display specific RG-I, enriched in α-(1-5)-arabinans and depleted in β-(1-4)-galactans, as well as low and highly methylesterified HG and RG-II, respectively (Orfila and Knox, 2000; Giannoutsou *et al.*, 2013; Paterlini *et al.*, 2022). PME subcellular localization around plasmodesmata, with additional cell wall remodelling enzymes, could cause the modification of pectin *in muro* (Morvan *et al.*, 1998; Fernandez-Calvino *et al.*, 2011). Such specific pectin patterns are suggested to regulate channel structure and symplastic cell–cell communication and might be critical for systemic virus infection (Chen *et al.*, 2000; Andika *et al.*, 2013). Further research is needed to fully understand the mechanisms involved in the formation and function of these pectin-specific microdomains.

## The case of epidermal pavement cell interdigitated growth

Arabidopsis leaf pavement cells possess a complex jigsaw puzzle-like shape that makes them an outstanding model for investigating cell polarity. The development of stomatal spiral cell complexes is regulated by a fluctuating auxin response gradient that is controlled by the precise localization of auxin transporters in young pavement cells (Grones *et al.*, 2020; Liu *et al.*, 2021). As these cells grow and form lobes, the pectin heterogeneity across their anticlinal curved cell walls becomes apparent. Specifically, RG-I side chain components galactan and arabinan are enriched on the convex side (neck or indentation region; Majda *et al.*, 2017) while HGs are less methylesterified in this region, where atomic force microscopy measurements show a softer wall. Conversely, highly methylesterified pectins are found on the concave side (lobed region), where the stiffness values are the highest (Majda *et al.*, 2017). Using a combination of super-resolution microscopy (3D-dSTORM) and cryo-scanning electron microscopy, Haas and colleagues revealed organized pectin nanofilament structures in the anticlinal wall that are able to swell at a low HG methylesterification degree, a conformation not observed in the periclinal wall (Haas *et al.*, 2020). This enrichment in low methylesterified HG and galactan/arabinan is observed in straight cell wall regions of young cells where lobes will likely appear, indicating a finely-tuned local regulation and the creation of microdomains prior to any observable growth asymmetry (Majda *et al.*, 2017).

**Fig. 2.**
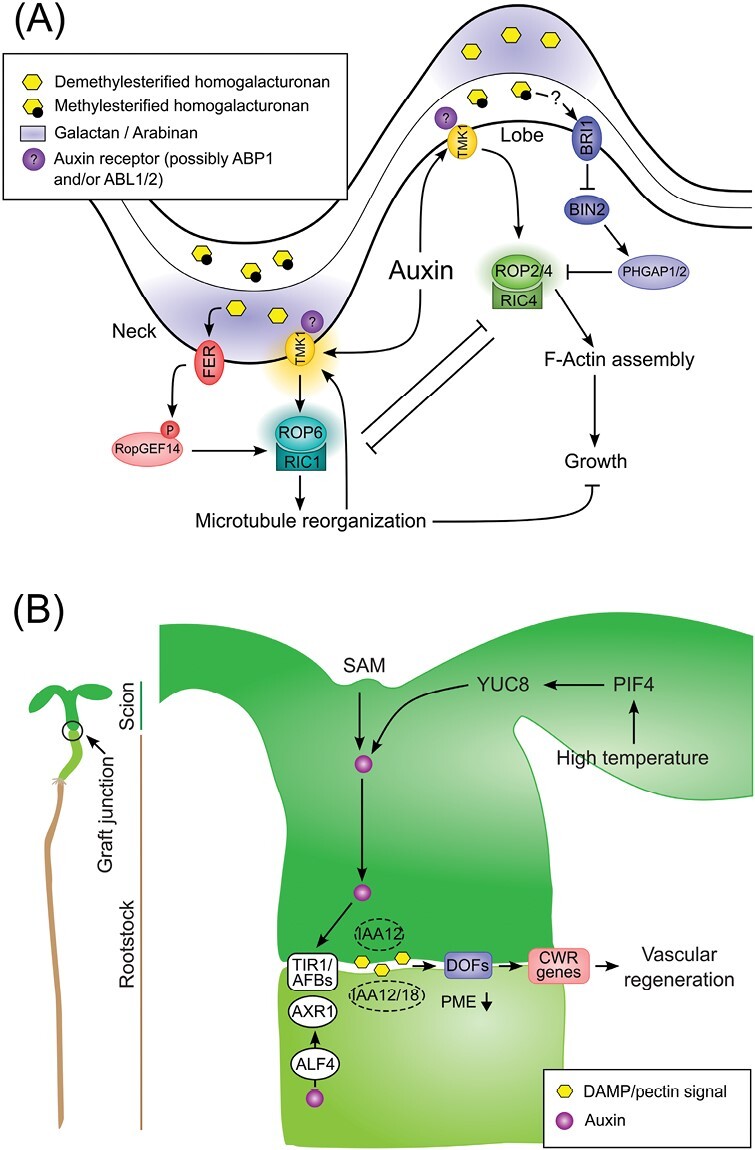
Overview of the auxin and pectin interplay in Arabidopsis epidermal pavement cell shape acquisition and hypocotyl graft interface. (A) Epidermal pavement cell shape working model. Cell surface auxin sensitive TMK1-based module, possibly involving the ABP1 extracellular auxin receptor or its close paralogue ABL, activates the ROP2/4-RIC4 module to trigger F-Actin filament assembly and lobe expansion. At the neck location, auxin promotes TMK1 nanoclusters activating the ROP6–RIC1 module to induce cortical microtubule reorganization and growth restriction. The convex side of the cell wall is enriched in galactan and arabinan pectin components and in demethylesterified homogalacturonans. Demethylesterified pectin are sensed by the extracellular domain of FER triggering phosphorylation of RopGEF14, in turn activating ROP6 signalling. At the neck location, high BRI1-mediated brassinosteroid signalling supresses BIN2-triggered phosphostabilization of PHGAP resulting in the activation of ROP2 signalling. On the concave side of the cell wall, the highly methylesterifified pectin signal is possibly transduced to BRI1 by an unknown mechanism. (B) Hypocotyl graft interface working model. Auxin is synthesized in the shoot apical meristem (SAM) or in the leaves under high temperature through a PIF4/YUC8 module, moves across the grafting interface, and elicits the TIR1/AFBs cascade degrading IAA12/18 and activating unknown ARFs to promote vascular regeneration. AXR1 and ALF4 are required in the root stock for vascular reconnection but not in the scion. Wounding at the graft interface produces a damage associated molecular pattern (DAMP), possibly resulting from pectin degradation products, and activates DOF transcription factors that regulate the expression of cell wall remodeling (CWR) genes to allow vascular regeneration. Reduced PME activity below the graft junction is a prerequisite for plant healing.

Pavement cell interdigitated growth is regulated by the Rho of plants (ROP) guanosine triphosphatase (GTPase) ROP2/4 and ROP6 signalling pathways (Fig. 2A). ROP2/4 and ROP6 have opposite effects on cell growth and interact with different proteins to control growth in different regions of the cell contour. At the neck region, ROP6 interacts with the ROP-interactive CRIB motif-containing (RIC) protein RIC1 to restrict growth and promote microtubule rearrangement, while at the lobe ROP2/4 interacts with RIC4 to promote cortical F-actin assembly and cell expansion (Fu *et al.*, 2005, 2009). Accumulating at the neck region and colocalizing with microtubules, the PLECKSTRIN HOMOLOGY GTPase-ACTIVATING proteins PHGAP1/2 inactivate ROP2. However, high brassinosteroid signalling can activate ROP2 in the lobe by suppressing BRASSINOSTEROID INSENTIVE 2-triggered phosphostabilization of PHGAPs (Fig. 2A) (Lauster *et al.*, 2022; Zhang *et al.*, 2022b). The demethylesterification of pectins in the cell wall at the neck region activates the transmembrane receptor-like kinase FER, which phosphorylates ROP guanine exchange factor 14 (RopGEF14) and activates ROP6 signalling (Fig. 2A) (Lin *et al.*, 2022; Tang *et al.*, 2022). The modification of pectins occurs prior to the initiation of lobes, indicating that the activation of FER could be one of the first elements to trigger the ROP/RIC signalling cascade. The activation of ROP2/4 and ROP6 antagonistic pathways in their respective lobe and neck locations is mediated by TMK-based auxin signalling at the cell surface, a mechanism that may involve ABP1 and/or ABL auxin receptors (Xu *et al.*, 2010, 2014; Yu *et al.*, 2022, Preprint). Pan *et al.* (2020) provide a comprehensive description of how auxin contributes to the formation of sterol and TMK nanoclusters at the plasma membrane leading to the local activation of ROP6 and the subsequent cortical microtubule reorganization that restrict the diffusive movement of TMK1 and ordered lipids. This process creates a self-amplifying ‘hot-spot’ for lobe outgrowth (Pan *et al.*, 2020). Auxin-mediated ROP6 nanoclustering was also observed in root cells, where it is dependent on an anionic phospholipid microenvironment (Platre *et al.*, 2019). It is unclear whether auxin–TMK-mediated ROP2/4 local accumulation also depends on distinct lipid nanodomains and membrane dynamics, and this requires further investigation.

Auxin-mediated proteolipid cluster formation plays a critical role in the formation of microdomains along the plasma membrane. Modification of the cell wall composition, particularly the pectin methylesterification status, affects protein dynamics, including PIN2 and PIN3 lateral mobility at the plasma membrane (Martinière *et al.*, 2012; McKenna *et al.*, 2019; Daněk *et al.*, 2020). Interestingly, loss of function of FER perturbs nanoscale dynamics of the immune receptor FLAGELLIN SENSITIVE 2/BRI1-ASSOCIATED RECEPTOR KINASE 1 (FLS2/BAK1) and perception of its small peptide ligand RALF is important for the nanodomain organization of BAK1 (Gronnier *et al.*, 2022). Besides the RALF peptide, locally demethylesterified pectins can bind to and activate FER (Feng *et al.*, 2018; Lin *et al.*, 2022), which, among other cell wall integrity sensor candidates, could potentially represent a link between the pectin matrix and protein clustering at the plasma membrane. Moreover, the relationship between FER and TMK1 in the light of auxin and pectin modification is worth investigating as they typically have opposite roles in plant growth and development. TMKs and FER could establish acidic or alkaline pH microdomains, respectively, by phosphorylating the common target, AHA2 (Haruta *et al.*, 2014; Lin *et al.*, 2021). Eventually, apoplast pH modification could alter the activity of pectin modifying enzymes, thereby introducing a feedback regulation mechanism to the model.

## How to sustain and pull the brakes on auxin action? The transcriptional control of auxin

Auxin exerts a critical transcriptional control to regulate cell growth by fine-tuning the expression dynamics of many key cell wall remodelling enzymes across diverse plant species (Laskowski *et al.*, 2006; Swarup *et al.*, 2008; Wei *et al.*, 2021; Zhang *et al.*, 2022c). MicroRNA167 (MIR167) targets *ARF6* and *ARF8* orthologues in diverse plant species (Barik *et al.*, 2015). In tomato, RNAseq analysis in MIR167 overexpression lines revealed down-regulation of many cell wall metabolism genes including pectin methyl transferase inhibitors, gluco-, manno- and galactosidases, UDP-glucosyl and -glucoronyl transferases, invertases, and β-1,3-glucan hydrolases (Liu *et al.*, 2014). Promoter analysis showed the presence of AuxREs in the promoter region of 109 out of a total of 686 mis-expressed genes including Solyc06g060170.2 (pectin lyase-like superfamily protein) (Liu *et al.*, 2014).

Pectin remodelling is a prerequisite for fruit ripening in both climacteric and non-climacteric fleshy fruits (Wang *et al.*, 2018). Reducing pectin degradation through silencing of pectate lyase or polygalacturonase genes delayed fruit ripening in tomato and strawberry (Santiago-Doménech *et al.*, 2008; Posé *et al.*, 2013; Uluisik *et al.*, 2016; Yang *et al.*, 2017; Wang *et al.*, 2019). The demethylesterification of HG and depletion in RG-I galactan side chains usually occurs during the ripening process (Wang *et al.*, 2018). Interestingly, the down-regulation of *Solanum lycopersicum ARF4* resulted in enhanced fruit firmness at the late ripening stage, an effect linked to differences in pectin fine structure and tissue architectural changes (Guillon *et al.*, 2008). In Japanese plum (*Prunus salicina* L.), auxin regulates pectin remodelling gene expression and accelerates ripening-related events such as fruit softening (El-Sharkawy *et al.*, 2016). In contrast, auxin delays ripening in pre-veraison grape berries (*Vitis vinifera*) and inhibits the transcription of several pectin-related genes including *PG*, *PECTIN LYASE* (*PL*), and *PME* (Dal Santo *et al.*, 2020). Accordingly, auxin treatment seems to delay pectin demethylesterification in this case, causing the berries to have stiffer wall and be less prone to cell expansion (Dal Santo *et al.*, 2020). Long non-coding RNA 7 (lncRNA7) and lncRNA2 play key role in modulating the cell wall defence response in cotton chromosome segment substitution lines when infected with the fungus *Verticillium dahliae* (Zhang *et al.*, 2022c). It was discovered that a lncRNA7–*GbPMEI13* module plays a positive role, while a lncRNA2*–GbPG12* module plays a negative role, in regulating disease resistance. The upregulation of lncRNA7 by pectin-derived oligogalacturonide promotes IAA accumulation and activates *GbPMEI13* expression, which inhibits *V. dahliae* mycelial growth and spore germination. ARF5 has been shown to induce *GbPMEI13* transcription. These findings suggest that GbPMEI13 has the potential to be a useful tool for improving disease resistance in plants (Zhang *et al.*, 2022c).

The transcriptional cascade initiated by ARF7 results in the accumulation of auxin in cells overlying the lateral root primordium (Stoeckle *et al.*, 2018). At this specific location, auxin activates the transcription of pectin modifying genes to accommodate the growth of the underlying primordium (Swarup *et al.* 2008; Kumpf *et al.*, 2013; Lewis *et al.*, 2013). Lewis and co-workers identified many pectin-modifying genes under the influence of auxin over a detailed and physiologically relevant time course during lateral root development (Lewis *et al.*, 2013). Equally, auxin controlled transcriptional responses are cell type dependent and fluctuate along the longitudinal axis of the primary root. Individual cells within four different root tissues in Arabidopsis exhibit specific response to auxin, with the epidermis being more susceptible to auxin-induced cell wall gene regulation (Bargmann *et al.*, 2013). Recently, it has been shown that in maize, there is a significant enrichment of transcripts associated with cell wall biogenesis in the root elongation zone following auxin treatment (McReynolds *et al.*, 2022). Interestingly, a pectin biosynthetic gene, *GALACTURONOSYLTRANSFERASE 15* (*GAUT15*), is transcriptionally regulated by auxin and required for root gravitropism (Lewis *et al.*, 2013). Quantitative root proteomics have identified another galacturonosyltansferase, GAUT10, that is post-transcriptionally regulated by auxin (Pu *et al.*, 2019). The ubiquitous role of GAUT10 in plant development implies a broad role for auxin-regulated pectin biosynthesis in many developmental contexts (Caffall *et al.*, 2009; Voiniciuc *et al.*, 2018; Guo *et al.*, 2021). A recent study on the role of GAUT10 in root apical meristem activity has identified a possible negative feedback loop on auxin signalling and metabolism (Dash *et al.*, 2023, Preprint). Pectin demethylesterification also appears to modulate auxin content during seedling development (Jobert *et al.*, 2022a, Preprint). Exogenously expressed fungal polygalacturonase increased pectin degradation and reduced auxin sensitivity in tobacco (Ferrari *et al.*, 2008). Furthermore, treatment with oligogalacturonide fragments, which typically result from polygalacturonase activity, produced a similar effect in Arabidopsis (Savatin *et al.*, 2011). Likewise, the down-regulation of a polygalacturonase involved in rootlet emergence in white lupin increased the expression of early auxin responsive *GRETCHEN-HAGEN 3* (*GH3*) genes (Jobert *et al.*, 2022b). Altogether, these results suggest that pectin backbone integrity affects auxin responses.

Despite the identification of various genes associated with pectin biosynthesis, the mechanisms that regulate their transcriptional activities remain mostly unclear. Recently, Xu *et al.* (2022b) showed that DE1 BINDING FACTOR 1 (DF1) is a key regulator of mucilage RG-I biosynthesis. DF1 and GLABRA2 (GL2) transcriptionally regulate the expression of *MUCILAGE MODIFIED 4* (*MUM4*) and *GALACTURONOSYLTRANSFERASE-LIKE5* (*GATL5*). Furthermore, it was shown that the expression of *DF1* and *GL2* is directly regulated by TRANSPARENT TESTA GLABRA2 (TTG2) (Xu *et al.*, 2022b). Interestingly, the *gl2* mutant displays hypersensitivity to auxin, and ARF1 and ARF2 interact with GL2 at the protein level (Llavata-Peris, 2013). Indeed, ARFs interact with many key transcription factors at the protein level (Cancé *et al.*, 2022). The BZR1–ARF6–PIF4/DELLA–ERF (BAP/DE) module has been shown to synergistically induce the expression of genes involved in cell wall biogenesis and organization, thereby integrating multiple hormonal and environmental signals during seedling morphogenesis (Bai *et al.*, 2012; Oh *et al.*, 2014; Liu *et al.*, 2018).

## The case of plant tissue regeneration during hypocotyl grafting

Plant grafting is a widely used technique in horticulture and scientific research, but the molecular mechanisms of graft formation and vascular reconnection remain poorly understood. Auxin gradient formation by the PINs plays a key role in regulating vascular development (Scarpella *et al.*, 2006). The scion and root stock tissues lose their asymmetric cell division, cell differentiation and gene expression patterns upon contact and develop vascular connections (Melnyk *et al.*, 2015). Auxin-mediated ABERRANT LATERAL ROOT FORMATION 4 (ALF4) activity promotes vascular connection by an inter-tissue communication process at the graft junction (Fig. 2B) (Melnyk *et al.*, 2015). Transcriptome analysis of the grafting interface revealed several genes associated with auxin signalling and cell wall remodelling during the early wound recognition and regeneration steps, respectively (Melnyk *et al.*, 2018). Recently, it has been shown that the perception of high temperatures in Arabidopsis leaves facilitates the regeneration of vascular tissues and formation of grafts in remote parts of the plant (Serivichyaswat *et al.*, 2022). Mutations in auxin biosynthetic genes (*YUCCA2/5/8/9*) or *PHYTOCHROME INTERACTING FACTOR 4* (*PIF4*) negate the regenerative capability at high temperature (27 °C) (Fig. 2B) (Serivichyaswat *et al.*, 2022). Furthermore, auxin activity and cell wall damage activate four DNA BINDING WITH ONE FINGER (DOF) transcription factor family genes, namely *HIGH CAMBIAL ACTIVITY2* (*HCA2*), *TARGET OF MONOPTEROS6* (*TMO6*), *DOF2.1*, and *DOF6*, to promote wound healing and tissue regeneration in Arabidopsis (Fig. 2B) (Zhang *et al.*, 2022a). The *dof* quadruple mutant exhibits changes in pectin composition and failed to trigger wound-induced pectin modification, suggesting a critical role for HCA2, TMO6, DOF2.1, and DOF6 in transducing cell wall signals in the early grafting events. A *PMEI5* overexpression line and the endo1,4-β-glucanase-deficient mutant *korrigan1* exhibit changes in the pectin and cellulose matrix, respectively, and display altered *HCA2* and *TMO6* expression (Zhang *et al.*, 2022a). Furthermore, alterations in the pectin and cellulose matrix can activate the wound-associated *ETHYLENE RESPONSE FACTOR 115* (*ERF115*) and *ANAC096* transcription factors, suggesting that cell wall damage induces a shared mechanism for wound perception and promotion of tissue regeneration (Zhang *et al.*, 2022a).

## Conclusion

Regulation of the pectin matrix by auxin occurs at the organ, tissue, cellular and subcellular levels and has crucial implications for how a plant responds to its environment. Auxin induces rapid variations in apoplastic microenvironment properties such as the pH value and concentration of important ions. This in turn locally affects the pectin backbone of the cell wall through the modulation of enzyme activity but is also dependent on the native pectin composition. Such cell wall microdomains should be seen as a dynamic continuum that links the cell wall, the plasma membrane, and cytosolic actors. Recent progress made in cell wall imaging using fluorescent probes and super resolution microscopy could help to paint a better picture of the pectin matrix structure in the near future. Simultaneously, advanced biophysical and biochemical analyses are required to establish a connection between wall mechanical properties and pectin chemistry. This could be facilitated by technologies such as atomic force microscopy and Brillouin microscopy coupled with high resolution LC-MS pectin dosage or Fourier-transform infrared spectroscopy. The precise mechanisms by which various PME enzymes operate with regards to de-esterification pattern and resulting mechanical outcomes are not yet fully comprehended. The regulation of different PMEs by auxin in different tissues may result in varying degrees of de-esterification and consequential effects. Additionally, pH and calcium sensors coupled to the expanding auxin molecular and genetic toolbox are allowing us to understand the very fast auxin cellular response. It will be interesting to investigate rapid modifications of pectin during these early auxin responses in a dynamic fashion. Transcriptional control of pectin remodelling by auxin activity has been extensively studied, but much less is known about the feedback exerted by pectin to the auxin machinery. Since cell wall structure is critical for plant adaptation to biotic and abiotic stresses, a balance between auxin and pectin remodelling needs to be reached. Understanding of feedback loops existing in this system and how they are connected to other molecular actors such as other phytohormones opens an exciting new area for future research (Box 1).

**Box 1. Outstanding questions**

- How can the generation of a structural interactome map and conducting a biochemical characterization aid in understanding the pH-dependent control of PME and PMEI, which belong to a large multigenic family, and shed light on the specific function of PME and PMEI in pH-dependent cell expansion?
- How much of the Ca^2+^ fraction required for rapid auxin-mediated growth inhibition originates from the pectin matrix? Can those pectin conformations be considered as a reservoir for Ca^2+^ transients observed during rapid auxin-induced growth inhibition in roots?
- Auxin rapid response signatures are too quick for transcriptional regulation, and therefore the cell wall must be predisposed to respond to changes in apoplast pH, whether it becomes more acidic or alkaline. Can the pectin matrix structure including the presence of readily active and pH sensitive remodelling enzymes determine the extent of auxin rapid response based on the cellular context?
- FERONIA and TMKs signalling outcomes on plant growth and stress response are well-documented but their direct interaction is still poorly described. Do these two receptors directly interact and to what extent do they share phosphorylation targets?
- How does local pectin heterogeneity occur along the straight wall before the initiation of a lobe in epidermal pavement cells? Does auxin play a role in establishing those pectin microdomains?
- How do pectin microdomains influence plasma membrane dynamics and the phospholipid environment?
- Auxin regulation of pectin remodelling genes is well described but much less is known regarding the impact of pectin integrity on auxin signalling and metabolism. A clearer picture that includes molecular actors is needed to better understand the feedback from the pectin matrix on auxin.

## Abbreviations

GalA: d-galacturonic acid
HG: homogalacturonan
PME: pectin methylesterase
PMEI: pectin methylesterase inhibitor
RG-I: rhamnogalacturonan-I
RG-II: rhamnogalacturonan-II
